# Influencing pro-environmental behaviors through visual arts: a scoping review of research designs and state of knowledge

**DOI:** 10.3389/fpsyg.2025.1712588

**Published:** 2025-12-04

**Authors:** Kelly Gbeve, Virginie Francoeur, Sophie Bernard, Louis Tanguay

**Affiliations:** 1 Department of Mathematics and Industrial Engineering, Polytechnique Montréal, Montreal, QC, Canada; 2 Québec Circular Economy Research Network (RRECQ), Montreal and Quebec City, QC, Canada; 3 Center for Intersectoral Studies and Research on the Circular Economy, École de Technologie Supérieure, Montreal, QC, Canada

**Keywords:** science-art projects, artwork, climate change, pro-environmental behavior (PEB), behavior change, methodology

## Abstract

**Background:**

Collaboration between scientists and artists is a growing trend driven notably by the need to take action against climate change. Consequently, the role of art is evolving to serve purposes beyond communication in the scientific community. With artistic projects increasingly becoming a form of intervention to influence pro-environmental behaviors, diverse methods are being deployed to assess their influence on behavior, yet little effort has been made to document coherently this emerging diversity of research designs and learnings.

**Objective:**

The aim of this study is to explore methodological approaches used to assess the effects of visual arts in promoting pro-environmental behaviors to fight climate change and determine whether some general trends can be observed.

**Methodology:**

We conducted a scoping review to identify empirical papers published between 2001 and 2024, from which 8 studies were selected. The selected papers assessed the influence of artistic intervention on pro-environmental behaviors and its defining variables.

**Results:**

We found six redundant attributes in research designs. Four of them (mixed-method approach, literature-based conceptual basis, longitudinal studies, baseline environmental profiles) can help support better research designs, while two others (supplementary material, co-creation) can enhance the influence of science-art projects on behavior. We also found that, among the diverse types of art forms (movies, festivals, exhibitions, murals, immersive installation), none were able to directly and significantly affect behaviors, but they could all affect environmental attitudes or awareness to some degree.

**Originality:**

This scoping review stands out from prior studies by highlighting the challenges and opportunities in assessing the influence of art on pro-environmental behaviors, as well as by exploring the role of visual arts for engaging the public in such behaviors.

## 1 Introduction

Climate change and its consequences have been documented in scientific literature for decades. International entities such as Intergovernmental Panel on Climate Change (2023) and scientific communities around the world are clear about the urgency to take action. However, despite the plethora of scientific information available, the limited actions currently undertaken at both local and global levels indicate that facts and science alone are not sufficient to trigger long-lasting changes in climate change-related behaviors (Allen et al., 2014). This might be due in part to the inaccessibility of scientific research and communication, either caused by a literacy or lexical barrier or by the subscribers-exclusive nature of many scientific journals (Chalmers, 1999; Dwivedi et al., 2024; Hayes, 1992).

The challenge of engaging the population in climate action is apparent in studies of pro-environmental behaviors (PEB), defined as the way in which one chooses to take actions that benefit the environment and avoid environmentally harmful actions (Lange and Dewitte, 2019). The literature in this field shows that engaging the population in PEB is a complex process (e.g., Ágoston et al., 2024; Francoeur and Paillé, 2022; Kollmuss and Agyeman, 2002). Indeed, while Andre et al. (2024), surveying nearly 130,000 participants in 125 countries, found that two thirds of people were willing to take action against climate change at a personal cost, other studies show that despite a widespread desire to adopt PEB, individual barriers limit their diffusion (Gifford and Nilsson, 2014; Kollmuss and Agyeman, 2002; Lucas et al., 2008). This divergence between intentions and behaviors is known in the literature as the “green gap,” defined as “the inconsistency between what the individual says regarding his/her growing concern about the environmental problems and what he/she does in terms of actions, behaviors, and contributions to lessen the consequences of these problems” (El Haffar et al., 2020: p. 38).

Given the above observation, cross-disciplinary approaches and solutions are necessary to address climate change challenges and help close the green gap. In this regard, collaboration between scientific and artistic communities has been increasing in recent years (Black et al., 2023). Art is already a favored method for scientific communication because it drives creativity, encourages exploration and resonates with different groups of people (Lesen et al., 2016), but art can also serve broader purposes, including inspiring action, driving cultural change and fostering community engagement (Global Leaders Institute, 2024). Contrary to scientific publications, art tends to be more accessible, being found in museums, schools, venues, parks, and is thus more likely to be part of people’s daily environment. Using art to communicate science provides many benefits: more accessible communication of scientific findings, increased awareness of research by experts and non-experts, greater impact and reach of science, and offering new perspectives to artists and scientists (Khoury et al., 2019; Lau et al., 2022; Zaelzer, 2020). Another advantage of art is its ability to convey and activate emotions, which is a highly relevant yet underexplored topic in PEB studies (Williamson and Thulin, 2022).

Despite the increasing popularity of science-art partnerships, these projects come with their own share of challenges (Gabrys and Yusoff, 2012). Challenges include the wide gap between researchers’ and artists’ expectations during collaborative projects, and limited recognition of the value-add of these forms of collaboration in the academic world. Methodologies in the context of science-art projects presents further challenges (Black et al., 2023). Since many disciplines, including environmental education, ecological economics, sustainability science and human resource management, are increasingly developing an interest in PEB, an abundance of methods and practices surrounding PEB are emerging, leading to a somewhat ill-defined field of study (Brick et al., 2024; Francoeur et al., 2021; Lange and Dewitte, 2019).

In this paper, we aim to tackle these challenges by providing insights into the diverse methodologies used for assessing PEB in contexts where art is proposed as a change agent, with a specific focus on visual arts. We also explore how art can actually contribute to changing behaviors. To do so, we conduct a scoping review of the relevant literature, addressing the following questions: (1) how can we evaluate the influence of visual arts on the adoption of pro-environmental behaviors? And (2) how can visual arts contribute to closing the green gap by triggering PEB adoption that tackle climate change challenges? The paper is divided into four sections. We first briefly review the literature on PEB and on the theory of planned behavior. Second, our methodology for the scoping review is described. Third, results concerning research designs and the influence of art on PEB are presented, and the main findings are discussed in the final section.

## 2 Conceptual background

### 2.1 Analyzing pro-environmental behaviors through the theory of planned behavior

In recent years, the study of pro-environmental behaviors (PEB) has attracted an increasing number of researchers from diverse disciplines (e.g., psychology, sociology, management or economics) (Tian and Liu, 2022). While definitions vary between publications, one of the most common comes from Kollmuss and Agyeman (2002, p. 240) who describe PEB as “behavior that consciously seeks to minimize the negative impact of one’s actions on the natural and built world.” This definition can be further extended with behaviors that aim not only at minimizing impacts, but at protecting and benefiting the natural environment and supporting environmental sustainability (Tian and Liu, 2022). The study of PEB emphasizes the agency of actors regarding their decisions to act or not to act in an environmentally and socially responsible manner (e.g., Ajzen, 2012; Yuriev et al., 2020; López-García et al., 2025).

**Figure 1:**
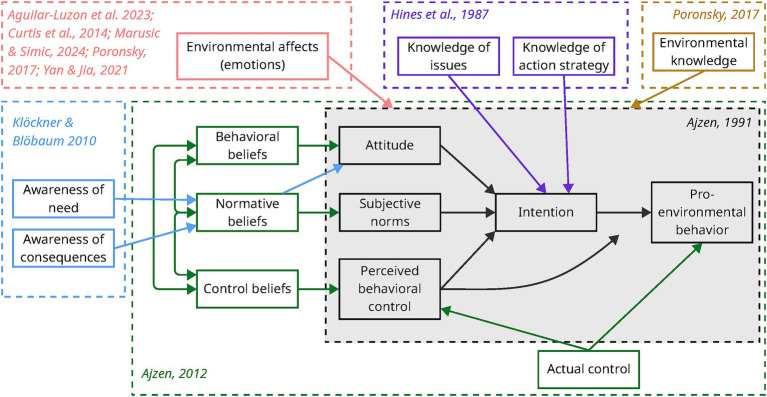
Insertion of emotions and environmental knowledge and awareness in the theory of planned behavior, based on a none-exhaustive account of the literature.

A range of approaches exist to evaluate PEB depending on the objectives of studies. A recent study that surveyed hundreds of environmental psychologists underlined the popularity of quantitative methods and self-report assessments as well as the frequent collaborations with governments and nonprofit organizations (Brick et al., 2024). Similarly, PEB can be analyzed using a plethora of models and frameworks. Among them, the theory of planned behavior (TPB) is one of the most widely used to study behavior in the literature (Yuriev et al., 2020). In an extensive literature review, Tian and Liu (2022) identified the TPB as a dominant model to explain PEB. In its original and simplest form, the TPB suggests that behaviors are influenced by the intention to act, which depends itself on: attitudes, whether favorable or unfavorable, towards a behavior; subjective norms, that is the perceived social pressure to act according to a behavior, and; perceived behavioral control, which refers to the perception of self-capacity to engage in a behavior (Ajzen, 1991). In a later publication, the author developed the model further to showcase how attitudes, subjective norms and perceived behavioral control depend on, respectively, behavioral beliefs, normative norms, and a combination of control beliefs and actual control (Ajzen, 2012) (Figure 1).

Despite its wide adoption, the TPM faces many critics because of its limited variables that offer a somewhat incomplete picture to explain behaviors, with variables such as knowledge, awareness, habits or emotions being poorly considered in the original concept (Cho and Walton, 2009; Klöckner and Blöbaum, 2010; Yuriev et al., 2020). However, the model makes up for these limitations through its flexibility, which allows authors to add variables as necessary based on their area of interest and research, or even to combine the TPB with other models to create new ones (e.g., Klöckner and Blöbaum, 2010). This flexibility is relevant for our paper, as detailed below.

### 2.2 Considering emotions, environmental knowledge and awareness through art in the TPB

Older models to study and explain behavior mostly emphasized the importance of knowledge and cognitive attributes (Kollmuss and Agyeman, 2002). For instance, the Model of responsible environmental behavior proposed that knowledge of environmental issues and action strategies could influence the intention to act in an environmentally responsible manner (Hines et al., 1987). By proposing a new approach to analyze behaviors, the TPB, in its first iteration, somehow neglected variables relating to knowledge (Ajzen, 1991). To overcome this shortcoming, many authors proposed to add environmental awareness and knowledge to the TPB (Yuriev et al., 2020). Among the most notable and comprehensive examples is a publication by Klöckner and Blöbaum (2010) who combined the TPB with the norm-activation model (NAM), the theoretical concept of habit and the ipsative theory of behavior. The authors proposed that awareness of the need to adopt PEB and of the consequences of one’s actions on the environment, acquired through environmental knowledge, could influence personal norms, and in turn environmental attitude and intention to adopt PEB (Figure 1). In that regard, art-based environmental education (Marusic and Simic, 2024), and more broadly, visual and performing arts (Curtis et al., 2014) can contribute to raising environmental awareness and communicating environmental knowledge.

Another notable limitation of the TPB is the absence of emotions from the model (Cho and Walton, 2009; Yuriev et al., 2020; Tian and Liu, 2022; López-García et al., 2025). Yet, emotional engagement is increasingly recognized as being at least as important as, if not more important than, cognitive engagement and environmental knowledge to influence behavior, and notably PEB (Kollmuss and Agyeman, 2002; Marusic and Simic, 2024; Poronsky, 2017). The literature presents many attempts to include emotions in the TPB, but they remain at best incomplete (Ho et al., 2024; Martini et al., 2023). Most attempts propose to include anticipated emotions as indirect predictors of behavior while ignoring immediate emotions, thus proposing once again a cognitive approach to including emotions in the process, as anticipated emotions relate to emotions that one believes would be triggered in a specific context or given a certain course of action (Ho et al., 2024). Ho et al. (2024) suggest including immediate emotions to integrate emotional responses experienced during decision-making regarding a given behavior, thus adding higher predictive capability to the TPB. The authors refer notably to a general bias towards short-term benefits rather than long-term ones, which influences behavior, especially when emotions come into play. This is notably the case when specific contexts or behaviors are perceived as risky or threatening. Beyond risk and threat perceptions, other emotions such as eco-anxiety, eco-worry, or on the positive side, nature connectedness, have all been explored in the literature, albeit through a very general perspective, and linked to the influence that they can exert on PEB (Figure 1) (e.g., Weber and Fiebelkorn, 2019; López-García et al., 2025; Yan and Jia, 2021; Yan et al., 2025).

Together with generating knowledge and raising awareness, emotions are one of the main venues through which art can influence behaviors (Tyler and Likova, 2012; Curtis et al., 2014; Poronsky, 2017; Raatikainen et al., 2020; Ma and Li, 2024; Marusic and Simic, 2024; Anasta et al., 2025; Finale, 2025). Therefore, integrating emotions into the TPB is especially crucial when studying the link between arts and PEB, but the literature remains unspecific regarding the processes that link art to PEB. The most prominent topic in this field explores how art creation at different stages of learning might positively influence environmental knowledge and attitude for school pupils and other publics (Tyler and Likova, 2012; Poronsky, 2017; Raatikainen et al., 2020; Marusic and Simic, 2024; Anasta et al., 2025; Finale, 2025). In all cases, art is shown to enhance situational emotions related to human-nature relationships. While studies regarding experiencing art as an audience are less common, they tend to show similar results, for example through the manifestation of awe (e.g., Ma and Li, 2024) or nature connectedness (Curtis et al., 2014).

## 3 Methods

We conducted a scoping review to answer our research questions. Scoping reviews are a suitable tool for investigating research trends on a specific topic and for identifying key messages and emerging knowledge (Munn et al., 2022). Our methodology mostly follows the guidelines of the PRISMA method (Moher et al., 2009), including three phases: (1) identification of the inclusion criteria applied to the articles considered; (2) data collection; and (3) data extraction and coding.

### 3.1 Inclusion and exclusion criteria

We considered empirical studies published in indexed peer-reviewed academic journals as well as academic theses, in English or French and produced between January 1st, 2001, and May 1st, 2024. We thus excluded non-empirical, theoretical or conceptual papers and reviews (Table 1). In terms of research topics, we solely included publications that evaluate the influence of artistic interventions on PEB and/or its variables (attitudes, intentions, etc.) among research participants. Interventions via extended reality were excluded because they are technology-focused rather than art-focused, as per their definition (Vasarainen et al., 2021). We focused only on visual art as the paper is part of a greater research project focused on the study of art presented in the context of exhibitions and festivals.

**Table 1:** Inclusion and exclusion criteria.

Characteristics	Inclusion criteria	Exclusion criteria
Temporal horizon	January 2001 to May 2024	Studies published before 2001 or after 2024
Design of study	Empirical (qualitative, quantitative and mixed method)	Theoretical, conceptual, reviews, non-empirical studies
Quality criterion	Indexed and peer-reviewed academic journals, master theses, and doctoral dissertations	Books, professional journals
Language of publication	English and French	All other languages
Outcomes measured	Influence of art on pro-environmental behaviors, or variables related to behaviors (attitudes, intentions, etc.)	Other behaviors not related to climate change, such as organizational behaviors
Intervention type	All art forms involving one or multiple artists	Research that does not involve artistic interventions or with a different focus (e.g., technology)

### 3.2 Data collection

We first skimmed through the relevant literature to identify relevant terms and formulate our search query, which is divided into four segments connected by the Boolean operator “AND” (Appendix A). These segments include terms related to: (1) art discipline (e.g., documentary or photography); (2) the environment (e.g., climate change or sustainability); (3) behavior (e.g., behavior or attitude); and (4) measurement methods (survey or questionnaire). Second, we conducted a literature search using Web of Science (English platform) and Érudit Database (French platform). The search in WoS (*n* = 590) and Érudit (*n* = 30) generated a total of 620 articles.

**Figure 2:**
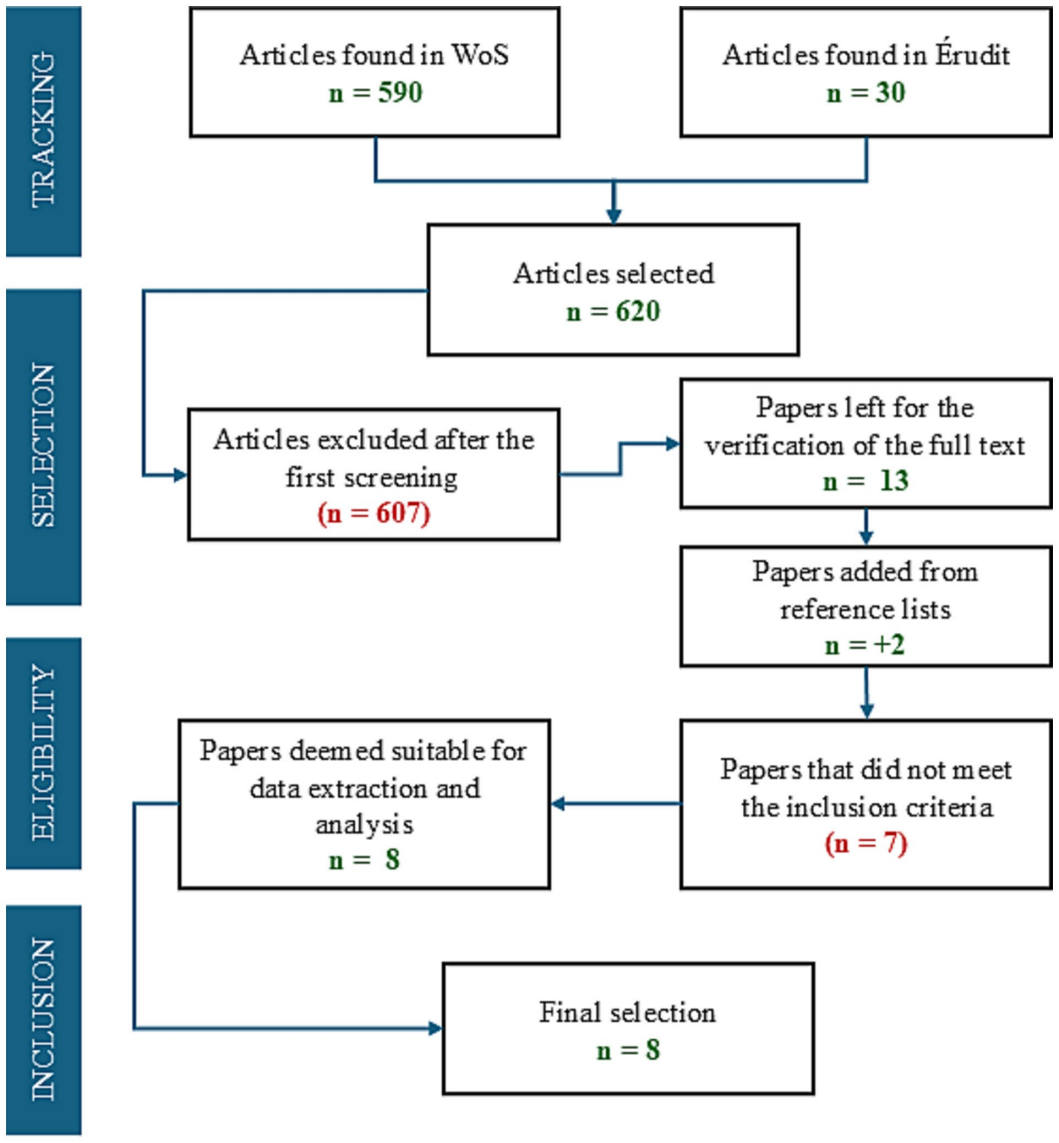
PRISMA flow diagram and selection of articles.

The articles (*n* = 620) were all uploaded to EndNote 20 software. The first screening was based on the articles’ title and abstract. We excluded articles that did not comply with the outcomes and intervention type criteria and ended up with a list of 13 articles (Figure 2). In addition, the bibliographic references of those articles were scrutinized to identify suitable articles based on their title, resulting in 2 additional articles added to the screening exercise. After reading the full texts and applying the inclusion and exclusion criteria, a total of 8 articles were retained: 6 coming from WoS, one from Érudit and one from the explored reference lists (i.e., Baldwin and Chandler, 2010). The latter article was not present in the original search because the abstract did not specify the use of measurement tools, which figure in our search query. Despite relatively few articles (*n* = 8) at the end of our screening process, we consider this number sufficient for the purposes of our scoping review, as it is in line with systematic and scoping reviews published in recent years in other emerging fields (see Ayassamy et al., 2024).

### 3.3 Data extraction and coding

We used Excel grids to structure the relevant information extracted from the articles. First, we categorized information regarding research design elements (objectives, sample characteristics, methodology). Second, we coded the results explored in the studies, using a mixture of deductive and inductive coding. Deductive coding was based on the TPB model and its variables (attitude, subjective norms, perceived behavioral control, intention and behavior) and permitted to establish a supported base ground to organize the information, while inductive coding allowed the necessary flexibility for other categories to emerge, especially regarding emotions. This led to the addition of two additional categories that we labelled “emotional activation” and “environmental awareness and knowledge.”

In cases where the data extracted from the studies were explicitly categorized by the authors as being part of one of our defined categories, we respected our own definitions and classification. Hence, in rare cases, the reported data vary slightly between our paper and their paper of origin (see Section 4.2).

## 4 Results

The results presented in this section were taken from the 8 reviewed articles. Main characteristics of each study can be found in Supplementary Table A.

### 4.1 Research design

#### 4.1.1 Creative processes

The involvement of researchers in the creative process and artists in the study design differ across the studies considered. We distinguish three categories of approaches. The first is co-creation, in which artists and researchers are involved from the beginning to the end of the project with high interaction between the two groups. This includes a photovoice project at the Floating Land festival (Baldwin and Chandler, 2010) and the Pollution Pods exhibition (Sommer et al., 2019). In the former case, the research project began with artwork creation, which consisted in a display of photographs with captions with the intent to communicate sense of place feelings and locally perceived consequences of climate change. Participants were asked to take pictures individually and were then split up into groups to select several photographs among the lot. These would then be presented to other participants and to a wider audience. Afterwards, participants completed a survey aimed at capturing the impact of the project on them.

The Pollution Pods exhibition (Sommer et al., 2019) results from a multidisciplinary research project called Climart based in Norway and overseen by a team of researchers in psychology, natural sciences and the arts. Michael Pinsky, who designed the “polluted pods,” worked in close collaboration with the research team to define the objectives of the artwork: “to raise awareness and increase engagement in the topic [of] climate change and air pollution […] with the ultimate goal of creating behavioral change” (Sommer et al., 2019, para. 5). Pods consisted of five connected geodesic domes with recreation of the air quality, albeit with safe substances, of five international cities, from dry cold to hot humid locations. The melding of arts and science is apparent in the artist’s involvement in the survey design and in the contribution of researchers in defining goals for the artwork and providing data to recreate the polluted pod environments.

The second category of creative process is mid-involvement, implying lesser interactions between artists and researchers during the creative process. This category includes two studies covering the various art forms exhibited at the Floating Land Festival (Marks et al., 2016) and the ArtCOP21 Climate Festival (Klöckner and Sommer, 2021). Although there was no direct collaboration between artists and researchers, pre- and post-event surveys were distributed. In the Floating Land case, artists and workshop hosts were surveyed together with festival attendees to gather viewpoints from people with varying levels of involvement in the events (Marks et al., 2016). The surveys were complemented by interviews with artists and field observations. Like the Pollution Pods from Sommer et al. (2019), the ArtCOP21 study fell under the umbrella of the Climart research group (Klöckner and Sommer, 2021). However, unlike the Pollution Pods, only the audience was surveyed, and there was no interaction between the research team and artists.

Lastly, the posteriori research category is defined by the absence of interaction between researchers and artists. Four studies fall into this category, reporting on the influence of documentaries (Girard, 2022; Hofman and Hughes, 2018), film (Howell, 2011) or murals (Schneller and Irizarry, 2014). In these cases, research on PEB took place well after the creation of the artwork, which is expected for films. As for the murals, the researchers built on the work of Grupo Tortuguero, a pioneer in the creation of sea turtle murals in Baja California Sur, Mexico, and with the purpose of engaging communities in behaviors beneficial to marine life. Even though most murals are created through participatory approaches, including collaboration with students, adults and other artists, the study by Schneller and Irizarry (2014) did not focus on those participatory processes but rather looked at the impact of sea turtle muralism in general.

#### 4.1.2 Art forms

**Figure 3:**
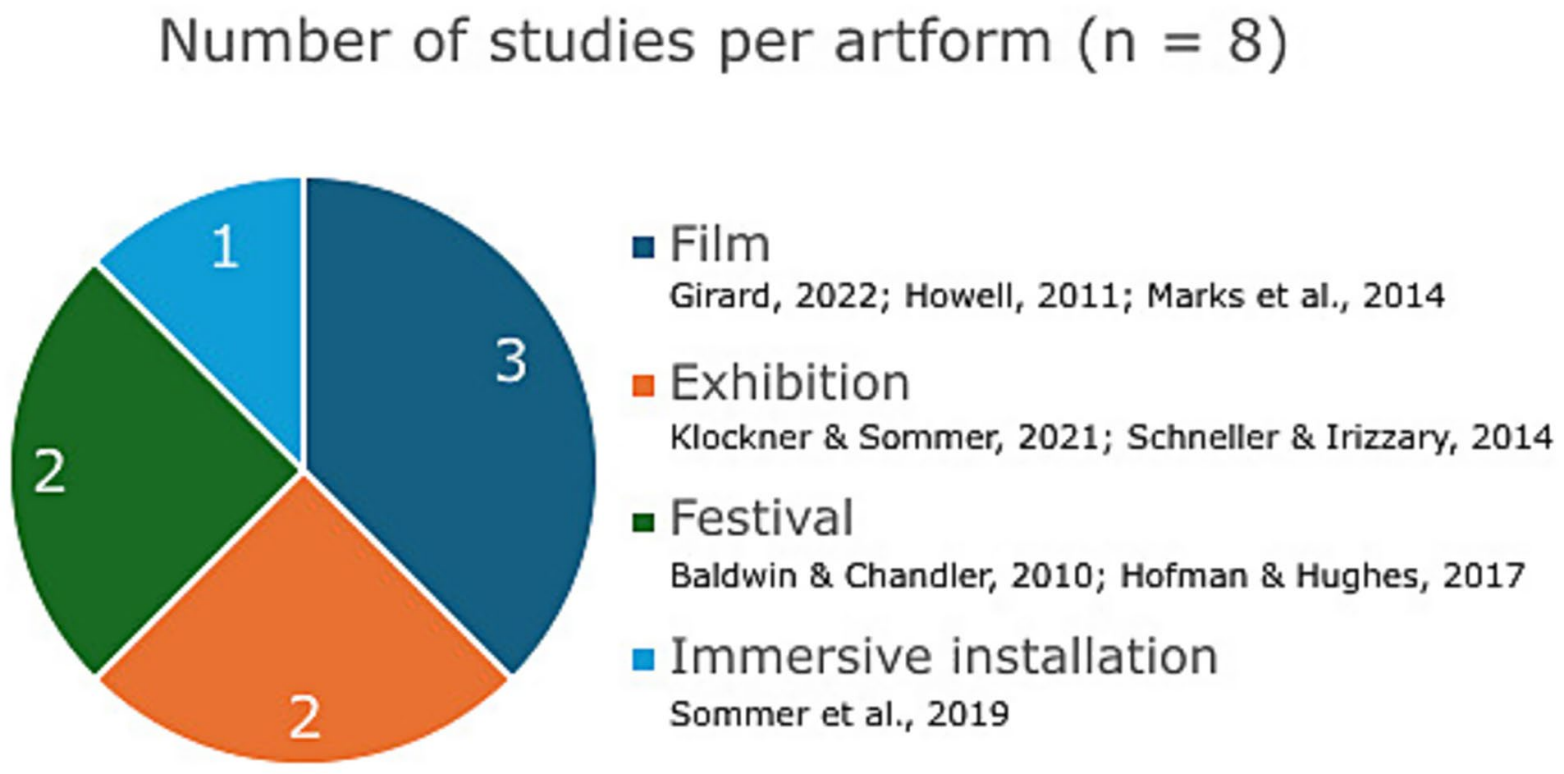
Distribution of art forms.

There is a fair representation of different visual art forms (Figure 3). Two studies analyzed documentaries (Hofman and Hughes, 2018; Girard, 2022). As one of the most common forms of scientific communication involving an artistic medium (Bieniek-Tobasco et al., 2019), we expected documentaries to be the prevalent art form among the publications reviewed. Girard (2022) focused on different persuasion methods (fear vs. solution-focused) used in documentary films, selecting 3 documentaries to do so: (1) Cowspiracy (2014), which centers on the environmental consequences of livestock farming; (2) Demain (2015), in which two protagonists present climate change issues and potential solutions from the perspective of agriculture, transport, the economy, governance and education; and (3) Les changements climatiques: le mur (2015), an episode from a television series depicting the relationship between energy-related challenges and climate change. Hofman and Hughes (2018) studied a documentary film about Australian marine environment conservation entitled The Sea and Me (2020). Howell (2011) focused on a different kind of film, studying the movie The Age of Stupid (2009), a combination of documentary footage and a fictitious portrait of the planet devastated by climate change in 2055.

Two studies covered the same environmental festival in Australia, Floating Land, albeit different editions. Baldwin and Chandler (2010) carried out a photovoice research project inviting participants to explore the threats of climate change and rising sea levels as part of the 2009 edition of Floating Land. Marks et al. (2016) examined the workshops, exhibitions, presentations and performances of the 2013 edition, including diverse art forms (e.g., sculpture, light and film projections, music and sound, poetic installations, dance). Similarly, 37 artworks from ArtCOP21, a joint event at the 21st United Nations Climate Summit in Paris, were at the center of Klöckner and Sommer’s (2021) study. Sommer et al. (2019) focused on Pollution Pods, an immersive installation of domes recreating the air quality of different cities around the world, while the remaining study by Schneller and Irizarry (2014) centered on an exhibition of sea turtle murals in Mexico, in relation to marine conservation.

#### 4.1.3 Methodologies and data collection

Three methodological approaches appear in the reviewed papers: qualitative (*n* = 1), quantitative (*n* = 3) or mixed-methods (*n* = 4). Schneller and Irizarry (2014) is the only qualitative study. The authors conducted semi-structured interviews and distributed open-ended questionnaires to explore the role of murals in complementing marine ecosystem management and environmental advocacy campaigns. Surveys were the favored method of measurement in both quantitative (Hofman and Hughes, 2018; Klöckner and Sommer, 2021; Sommer et al., 2019) and mixed methods (Baldwin and Chandler, 2010; Girard, 2022; Howell, 2011; Marks et al., 2016) studies. Likert scales were widely used, with 7-point Likert scales being the most common (Hofman and Hughes, 2018; Howell, 2011; Klöckner and Sommer, 2021; Sommer et al., 2019), followed by 5-point Likert scales (Baldwin and Chandler, 2010; Girard, 2022). Statements within the scales vary between publications, with the most measurement item being “agree/disagree,” used in 4 studies (Howell, 2011; Hofman and Hughes, 2018; Sommer et al., 2019; Klöckner and Sommer, 2021). Multiple-choice questions were rarely used, except in Howell (2011) who evaluated behaviors using either “not doing/not done,” “doing/done, because of film,” or “doing/done not because of film” categories. Finally, open questions were used in the mixed-method studies to complement most of the quantitative studies since they offer complementary insights into participants’ responses.

Self-report assessments through paper or online surveys were the preferred approach in all studies, as is commonly the case for PEB assessments due to their low cost and scalability (Lange and Dewitte, 2019). Surveys were completed by participants on site in all studies but one (Girard, 2022), but follow-up surveys were completed online since they took place several days or weeks later. In addition to surveys, Girard (2022) also used a technological tool, ReaQtor, to capture real-time feedback from participants. As they were viewing the documentary films, participants could indicate if they felt “motivated” or “disinterested” by specific parts of the film.

Most of the reviewed studies are cross-sectional (Baldwin and Chandler, 2010; Marks et al., 2016; Schneller and Irizarry, 2014; Klöckner and Sommer, 2021; Sommer et al., 2019), except for those focusing on films which are longitudinal (Girard, 2022; Hofman and Hughes, 2018; Howell, 2011). In the latter cases, follow-ups occurred either 4 weeks (Girard, 2022), 6–8 weeks (Hofman and Hughes, 2018) or 10–14 weeks (Howell, 2011) after film visioning. On a side note, Sommer et al. (2019) did plan to carry out a longitudinal study to capture behavioral change via an online platform, but the low participation rate (2% for exhibition visitors and 0% for the control group) prevented their attempt (Table 2).

**Table 2:** Participation rate in post-event survey and follow-up phase.

Reference	Art form, project	Pre-event participants	Follow-up participants	Time lapse
Girard (2022)	Climate change documentaries, Cowspiracy (2014), Demain (2015), Les changements climatiques: le mur (2015)	24	22 (92%)	4 weeks
Hofman and Hughes (2018)	Documentary, The Sea and Me (2020)	182	84 (46%)	6–8 weeks
Howell (2011)	Movie, The Age of Stupid (2009)	241	162 (67%)	10–14 weeks
Marks et al. (2016)	Environmental art festival, Floating Land (residents)	60	36 (60%)	Shortly after event
Sommer et al. (2019)	Immersive installation, Pollution Pods (Trondheim)	Participants: 1016	24 (2%)	Shortly after event
		Control: 415	4 (0%)	Shortly after event

### 4.2 Linking art to PEB

The reviewed studies captured the influence of art on PEB in several manners, as reflected in the categories created through our coding process. As mentioned in section 3.3, we based our coding process on the TPB variables (attitude, subjective norms, perceived behavioral control, intentions to behave and PEB), to which we inductively added two categories: emotional activation, and environmental awareness and knowledge. It is worth noting that, despite the existence of the “subjective norms” category, no findings extracted from the reviewed papers could be associated with it, for reasons further explained in the Discussion.

Below are the definitions used to describe our results, derived from Ajzen (1991) unless stated otherwise:

- Emotional activation refers to the emotions, whether positive or negative, aroused in participants after a stimulus (in our case, the artworks) (Junça-Silva et al., 2018).
- Environmental awareness and knowledge refer to sensitivity towards, and understanding of, the natural environment and associated issues (Marusic and Simic, 2024).
- Attitudes represent an individual’s positive or negative viewpoint regarding a specific behavior or PEB.
- Perceived behavioral control is the level of control that an individual believes to have for engaging in a specific behavior.
- Intentions refer to the prospected behavior that individuals expect to exhibit in the future.
- Pro-environmental behaviors (PEB) are concrete observable actions that benefit the environment, whether through inhibiting harmful behaviors or adopting beneficial behaviors towards the natural environment.

#### 4.2.1 The influence of art on PEB

Main findings reported in the eight reviewed studies are distributed across all described categories (Tables 3–8; detailed categorizations of measurement items appear in Supplementary Table B for quantitative and mixed-method studies). PEB variables most observed to be influenced by art are attitudes and intentions, with five studies out of eight highlighting influences for each of these categories. The four other variables were each found to be influenced by art in only three studies.

**Table 3:** Main findings regarding emotional activation.

Study	Findings
Klöckner and Sommer (2021)	Reflecting on the artwork led to both negative and positive emotions among participants. Stronger emotional responses led to stronger support for climate policy, especially in the case of negative emotions.
Marks et al. (2016)	38% of participants agreed with the statement “I feel guilt about the state of the environment,” but it is unclear whether this was due to emotional activation during the event or not.
Sommer et al. (2019)	Sadness, helplessness, and anger had the strongest influence on intentions to change behavior (only significant predictors).

**Table 4:** Main findings regarding environmental awareness and knowledge.

Study	Findings
Baldwin and Chandler (2010)	56% confirmed that the Photovoice project had raised their awareness of climate change. However, 71% of those surveyed revealed that the photovoice project reinforced their views, with several respondents commenting that they already had a high level of awareness of climate change.
Hofman and Hughes (2018)	77% of participants reported that their knowledge of marine conservation had increased after viewing the documentary The Sea and Me (2020), and an increase in environmental awareness (measured by the item “We are part of the threat to marine environments”) was also observed.
Schneller and Irizarry (2014)	Most participants (students and adults) reported that the murals had increased their awareness of environmental issues.

**Table 5:** Main findings regarding attitudes.

Study	Findings
Baldwin and Chandler (2010)	69% felt encouraged by the photovoice project to take action against climate change.
Hofman and Hughes (2018)	A significant percentage^a^ of participants showed an increase in positive attitudes toward marine conservation actions as a result of viewing The Sea and Me (2020) documentary.
Howell (2011)	Agreement with the statement “I feel motivated to try to do something about climate change/global warming” increased to 95.8% after viewing the movie (from a 90.2% baseline). However, that result decreased to 90.4% during the follow-up phase, indicating that the effect of the film alone did not last long term.
Klöckner and Sommer (2021)	Participants with weak initial environmental attitudes were more likely to show a strong connection between reflection on the artwork and support for climate policy than participants with strong initial environmental attitudes, possibly because the latter were already concerned with such issues and already supported climate policy.
Schneller and Irizarry (2014)	Most participants (students and adults) reported that the murals had increased their positive attitude towards the natural environment

a The exact percentage cannot be provided given that our coding differ from the authors’ original coding and we lack access to the original data.

**Table 6:** Main findings regarding perceived behavioral control.

Study	Findings
Girard (2022)	Perceived behavioral control did not appear to be affected by any of the three documentaries, whether directly after the viewing or in the follow-up phase.
Hofman and Hughes (2018)	An increase in perceived behavioral control (measured by the item “There is a lot I can do to help protect marine environments”) was expressed by participants after viewing the documentary The Sea and Me (2020).
Howell (2011)	The viewing of The Age of Stupid (2009) increased participants’ sense of agency in fighting climate change, but the effect had worn off in the follow-up phase. Key barriers were identified for participants who responded that they wanted to do more in relation to different categories: Raise awareness/lobbying: 70.7% identified lack of time Home energy use: 51% identified cost Travel: 46.8% identified the lack of options Food: 42.4% identified the lack of options

**Table 7:** Main findings regarding intentions.

Study	Findings
Girard (2022)	The initial intentions of participants, who all agreed or strongly agreed with the item stating that they intend to adopt a more environmentally responsible behavior, did not show significant change after viewing the documentaries.
Hofman and Hughes (2018)	72% of participants indicated that they intended to participate in marine conservation activities as a result of viewing the documentary.
Marks et al. (2016)	In the audience survey, 41% of participants indicated that they intended to change their environmental behavior as a result of attending the Floating Land festival. Of these, just over half (24% of audience participants) attended the festival without initial environmental intentions, and some participants (unspecified number) mentioned that they did not intend to change because they believed to have already adopted PEB. 44% of respondents to the Boreen Point resident survey stated that they had wanted to do more for the environment since attending the Floating Land festival.
Schneller and Irizarry (2014)	Some participants indicated that observing the murals may encourage them to adopt PEB, particularly in relation to reducing pollution and the illegal consumption of sea turtles, but none of the adults reported that they would adopt fishing techniques to reduce the accidental capture of sea turtles.
Sommer et al. (2019)	The results showed that changes in intentions to address pollution and climate change were statistically significant, although minor. Participants who already expressed strong intentions entering the pollution pods showed lesser intentions to change their behavior.

**Table 8:** Main findings regarding behaviors.

Study	Findings
Girard (2022)	Documentaries that used fear and/or solutions for persuasion purposes had little influence on PEB (e.g., less meat consumption in a few participants).
Hofman and Hughes (2018)	Documentaries have a marginal impact on long-term behaviors since the results show that 6–8 weeks after the viewing, behavior measurements declined and, in some cases, were even lower than the baseline (pre-viewing). PEB decreased notably for participants of the control group who did not receive further support after the viewing (through Facebook, help sheets or both), while PEB remained at the same level or slightly increased in the experimental groups (who were given support) for PEB such as recycling, choosing sustainable fish, putting oil down the drain, using green bags, conserving energy and using minimal packaging
Howell (2011)	60% of respondents stated that at least one action they are taking (or doing more of) was because of viewing the film. These actions included raising awareness of climate change among people they know, lobbying politicians, and reducing their carbon footprint.

According to Marks et al. (2016), Sommer et al. (2019), and Klöckner and Sommer (2021), art is able to activate emotions, with an apparent prominence for negative emotion activation such as guilt or anger. Art is also able to raise environmental awareness and develop environmental knowledge if designed for such a purpose (Baldwin and Chandler, 2010; Schneller and Irizarry, 2014; Hofman and Hughes, 2018), although participants in environmentally oriented art or exhibitions might already possess a strong environmental awareness (Hofman and Hughes, 2018), hence reducing the potential influence of art on them. Positive environmental attitude generally increased with exposure to art (Baldwin and Chandler, 2010; Howell, 2011; Schneller and Irizarry, 2014; Hofman and Hughes, 2018; Klöckner and Sommer, 2021). However, in a follow-up survey carried out 10–14 weeks after viewing documentary film, Howell (2011) found positive attitudes to be back to the initial, pre-viewing level.

Perceived behavioral control is less consensual, with Girard (2022) reporting no influence on this variable by the documentaries, either right after watching them or 4 weeks later, while Hofman and Hughes (2018) and Howell (2011) found a positive influence. However, the latter author also found that many perceived barriers remained after watching the film, leading to lower perceived behavioral control in participants. Studies that reported the influence of art on intentions to adopt PEB mostly found a small effect on those intentions in a fraction of participants (Hofman and Hughes, 2018; Marks et al., 2016; Schneller and Irizarry, 2014; Sommer et al., 2019), except for Girard (2022) who observed no effect, and Schneller and Irizarry (2014) who observed that when asked about a more specific action to protect marine life, fishers showed no intention to modify their behavior accordingly.

The influence of art on behavior could only be assessed in longitudinal studies, hence in studies using documentaries or film as an art form. Girard (2022) and Hofman and Hughes (2018) found that documentary movies only had a marginal effect on PEB several weeks after the viewing (respectively 4 weeks and 6–8 weeks). However, Howell found a positive effect on PEB 10–14 weeks after viewing the documentary in 60% of respondents.

Apart from the influence of art on PEB variables, some authors were also interested in studying how PEB variables influence each other in participants. Most notably, Klöckner and Sommer (2021) linked strong emotional activation with stronger support for climate policy, linking directly emotional responses to a more positive environmental attitude. Further, Marks et al. (2016) measured attitudes as a predictor of intentions, observing that individuals who showed a strong sense of responsibility and optimism towards the environment had greater intentions to adopt PEB. They also found the same to be true regarding individuals who believed that their actions can have a positive impact on climate change, or in other words, individuals with strong perceived behavioral control.

#### 4.2.2 Artwork effectiveness

Beyond the survey processes focusing on participants’ perspectives, artwork effectiveness to influence PEB was assessed in three studies (Girard, 2022; Klöckner and Sommer, 2021; Marks et al., 2016). However, the only notable findings come from Klöckner and Sommer (2021) who argue that some artworks features were strongly correlated to emotional responses and to higher levels of reflection. More specifically, their findings suggest that 23% of the variance in positive emotions and 20% of the variance in negative emotions were attributed to the artwork features such as color, personal identification, size, etc. Furthermore, artworks that challenged social norms or included personal elements with which viewers could identify led to greater reflection on the art work.

## 5 Discussion

### 5.1 Learnings from research designs

The range of research designs included in this scoping review (*n* = 8) provide relevant insights for future research interested in the influence of art on PEB. We identify six particular design attributes that merit attention (Table 9). The first four can arguably contribute to developing more comprehensive research projects. They are mixed-method study design, literature-based variables, longitudinal studies and establishing baseline environmental profiles.

**Table 9:** Attributes to consider for PEB assessments in an artistic context.

Attributes	Description	Examples
Mixed-method study design	A mixed-method approach can help gather more in-depth information that is difficult to capture through Likert scale measurement items.	Howell (2011) captured the main takeaways from participants and was able to determine that the “need to take action” was the most frequent theme.
Literature-based variables	Use of the existing literature on behavior change and PEB to select appropriate variables and measurement items.	Girard’s (2022) holistic approach evaluates the entire behavior adoption process.
Longitudinal studies	Planning longitudinal studies beforehand and recruiting participants accordingly can help obtain higher participation rates for follow-up surveys.	Girard’s (2022) achieved a participation rate of 92% through prior recruitment of participants.
Baseline environmental profiles	Consideration of baseline environmental beliefs, attitudes, intentions and behaviors when creating the work and collecting data.	Marks et al. (2016) distinction between behavior-changers and non-behavior-changers might have been biased because of a lack of baseline environment profiles.
Co-creation	Integration of artists and researchers at various stages of the process, ensuring consistency between the artwork and the study design with the target audience and the targeted PEB.	Two projects were co-created by researchers and artists (Baldwin and Chandler, 2010; Sommer et al., 2019).
Support material	Use of support material to maintain the impact of the artwork after the event, serving as a reminder and a platform for exchange.	Hofman and Hughes (2018) provided support such as fridge magnets and a list of actions to take.

Although quantitative studies dominate the PEB literature in environmental psychology (Brick et al., 2024), mixed-method studies were most common in our scoping review. Girard (2022) noted that qualitative answers were richer and could be used to a greater extent through semi-structured interviews with participants. Sommer et al. (2019) noted that the measurement scale used to capture the strength of the impact of Pollution Pods on participants’ intentions was constraining due to its quantitative nature, thus pointing to the greater richness of data that can be brought about by a mixed-method approach.

Considering the existing literature on behavioral change and PEB is another key attribute and can support the selection of appropriate variables and measurement items to design a comprehensive research project. For example, Girard (2022) took a holistic approach by assessing the overall behavior adoption process using four methods built on previous theoretical frameworks found in the literature. Accordingly, the survey design considered intentions, perceived behavioral control, awareness of consequences, PEB, and self-perceived behavioral change. Such an approach can notably help understanding the pathways through which a studied object, such as art in our case, can influence intentions and behaviors. While this might seem evident at first glance, only half of the reviewed papers (Girard, 2022; Klöckner and Sommer, 2021; Marks et al., 2016; Sommer et al., 2019) engaged significantly with the existing literature on behavior to build their conceptual background. Further, none of the papers provided adequate definitions for the terminology used, even in the case of ambiguous terms such as “attitude,” leading to somewhat confused conclusions.

When studying the direct influence of art on behaviors, longitudinal studies are an essential attribute of research design, but ensuring a high participation rate for follow-up surveys remains a challenge. One of the hypotheses of the Pollution Pods study could neither be confirmed nor denied because of a low participation rate in the post-event surveys (2%), possibly due to a time-consuming process (Sommer et al., 2019). Hofman and Hughes (2018) faced similar issues, to the surprise of the authors. While the pre- and post-event surveys were completed during a class, the follow-up surveys were sent in an electronic format. This shift in strategy may explain why only 46% of students participated in that phase. The most successful study was Girard (2022), with a 92% participation rate, probably due to the fact that participants were recruited for the study prior to viewing the documentary film, whereas two of the other longitudinal studies intercepted participants at movie theaters (Howell, 2011) or during the exhibition (Sommer et al., 2019). One notable tactic used in one study (Howell, 2011) was to offer a ₤10 voucher as an incentive to participate in the follow-up survey, which might have contributed to the 67% participation rate.

Baseline beliefs, attitudes, intentions and behaviors associated with environment-related topics influences the outcome of an intervention aiming to shift behaviors. For instance, individuals with stronger environmental attitudes may, on the one hand, have a greater understanding of the subject or show greater intentions to change or, on the other, consider that they are already doing enough or as much as possible. As an example, Marks et al. (2016) distinction between behavior-changers and non-behavior-changers might have been biased because of a lack of baseline environment profiles, and non-behavior-changers might in fact include participants who were already engaged in PEB before the study. It is thus relevant to consider participants’ baseline environmental profiles, and to aim for a higher heterogeneity as much as possible. Indeed, in a relatively homogenous group, the main observation relates to the group represented in the sample (El Haffar, 2022). Therefore, the conclusions drawn are biased toward that group and overlook the diversity of a population. This reduces the generalizability of the study and its potential to serve as a decision-making tool for policymakers.

The two additional research design attributes worth discussing are less connected to the quality of design than to design choices that come with both positive aspects and limitations. These are co-creation of the science-art project by researchers and artists, and the provisioning of support material. Co-creation can support better integration of the study objectives with the artistic process, leading to higher consistency and facilitating measurements. Co-creation has an especially important influence if participants are invited to act as the artists themselves, as seen in Baldwin and Chandler (2010) with the photovoice method, thus leading to a much deeper engagement with the process and in turn, to greater influence on PEB (Carlson et al., 2006). Support material, when provided between the initial and follow-up surveys in longitudinal studies, can directly perpetuate attitudinal changes that resulted from art exposure, as seen in Hofman and Hughes (2018). In that later case, provided material included a magnetic help sheet, pictures, a list of actions to undertake as well as a Facebook group to exchange ideas on the topic of marine conservation. While both co-creation and support material have advantages and might lead to more positive results, they do not reflect real-life contexts where people would simply be exposed to art spontaneously without the involvement of researchers. These limitations are important to understand as results from such studies are less generalizable.

### 5.2 The contribution of art to PEB

The reviewed studies highlight the different roles that art can play in affecting PEB and its variables of influence. Authors report how art can influence emotions, knowledge and awareness, attitudes, and to a lesser extent, perceived behavioral control and intentions to adopt PEB, showcasing art as a potential contributor to PEB adoption. However, as stated by Sommer et al. (2019), an art experience alone cannot be expected to result in behavioral change. Indeed, the three studies that measured the direct influence of art on PEB (Girard, 2022; Hofman and Hughes, 2018; Howell, 2011) concluded that the artworks under study had limited impact on participants’ behaviors. Therefore, art alone may not be a sufficient tool to bridge the green gap, at least not as a single intervention.

A common shortcoming among the reviewed papers is the lack of a comprehensive understanding of the processes through which art affects participants. While authors studied attitudinal or intentional changes, the process behind those changes remains fuzzy. Even in papers studying emotions (Klöckner and Sommer, 2021; Marks et al., 2016; Sommer et al., 2019) or environmental knowledge and awareness (Baldwin and Chandler, 2010; Hofman and Hughes, 2018; Schneller and Irizarry, 2014), the links between these variables and other PEB variables were vaguely explored at best. The only notable exception comes from Klöckner and Sommer (2021) who, linking strong emotional activation with stronger support for climate policy, were able to confirm that emotions could have a significant influence on attitudes, at least in the short-term, thus bringing more precision to broad observations found in the literature and stating that emotions can affect PEB as a whole (e.g., Aguilar-Luzón et al., 2023; Curtis et al., 2014; Marusic and Simic, 2024; Poronsky, 2017; Yan and Jia, 2021) (Figure 1). Such exercise allows further disentangling of complex processes of behavior, and more efforts in the same vein could certainly support a better understanding of how art affects PEB. Furthermore, as observed by Klöckner and Sommer (2021) and Sommer et al. (2019), activation of negative emotions appears to exert a greater influence on PEB variables than positive emotions. This is in line with trends observed in the literature (e.g., Cho and Walton, 2009; López-García et al., 2025) and can be explained at least partially by how environmental issues can be perceived as risks or life-threatening situations (Ho et al., 2024).

Of course, art also has limitations in how it can affect PEB, and one of these comes from emotions. Emotional activation, as we defined it for the purpose of our article, mostly relates to the activation of immediate emotions, defined in section 2.2 as emotions experienced in the very moment a risk is perceived, or an action or decision is taken (Ho et al., 2024). The fact that art activates immediate emotions limits its potential long-term influence on PEB, as observed in the reviewed longitudinal studies (Girard, 2022; Hofman and Hughes, 2018; Howell, 2011). Only in Hofman and Hughes (2018) was art able to influence PEB or its variables on a longer-term, and most probably because of the authors’ recourse to support material. Otherwise, art exerted only a punctual influence on any PEB variables. A possible cause is that the activated immediate emotions mostly motivate what is referred to as hedonic goals in the Goal framing theory, which refers to actions or intentions motivated by the intent to improve one’s current feelings (Lindenberg and Steg, 2014; Prior, 2022). Thus, after exposure to a particular art form that activated their emotions, participants quickly went back to their original perceptions, attitudes, intentions or behaviors once emotions had worn off. Yet, for PEB to be adopted on a longer-term, PEB need to be motivated by other types of goals. Gain goals, where intentions and actions are motivated by the benefits procured by a particular choice, can certainly support PEB as well, but they might be short-lived if one stops receiving the pursued benefit and thus retract from PEB (Prior, 2022). Normative goals, supporting actions that are undertaken to conform to social norms and rules, might help further strengthen behaviors in the long-term. Normative goals can themselves be influenced by values and subjective norms (Steg et al., 2014), which, in the latter case, were not found to be influenced by art in any of the reviewed studies, as stated in section 4.2. This is not surprising given the fact that most studies were based on punctual exposure to art forms. Yet, it might also explain why among the three longitudinal studies, the one study that provided support material to participants (Hofman and Hughes, 2018) reported at least some longer-lasting changes in participants’ behavior. The support material might have contributed to a perception of standardization of PEB among participants. This observation points to the importance of establishing long-term trends to influence behavior, and art can certainly play a role in establishing those cultural shifts towards standardization of PEB. Thus, while art exposure, when analyzed as individual case studies, might not seem to be the most effective manner through which PEB can be encouraged, continued exposure to art associated with environmental topics can certainly contribute to normalizing PEB as part of a bigger trend.

### 5.3 Limitations and future research avenues

Several limitations must be mentioned for the present paper. First, regarding the methodology, the number of articles considered is relatively small for a scoping review, which limits the trend analysis since most projects had a unique take on how to research and measure the influence of art on PEB. This is due, in part, to the scarcity of research projects bridging arts and science. This might have been solved to a certain extent had we decided to include art forms beyond visual arts (e.g., literature, music) or terms related to the biodiversity crisis in our search terms, which should be considered in future literature reviews to broaden the perspective on our topic. Further, extending the time period covered in future literature reviews could also help in including more studies.

Second, searching solely in the scientific literature for science-art projects closed the door to other initiatives in which artists might undertake artistic projects to foster PEB or raising awareness of climate change without being linked to a research project. Our Connection to Water exhibition in the United Kingdom (Royal Museums Greenwich, 2025), American artist Zaria Forman’s pastel drawings (Forman, 2025), the eARTh – Artists as Activists exhibition in the United States (MutualArt Services, 2025), and the ZERO [waste] Exhibition in Canada (Francoeur and Bernard, 2024) are some examples. Conducting qualitative analyses with artists and researchers who have carried out this type of project may be relevant to broadening the scope of potential science-art projects to study. It could also be useful to look more closely at funding programs such as Horizon Europe (European Commission, 2025) that can support this sort of science-art partnership.

Third, in many of the reviewed studies (e.g., Baldwin and Chandler, 2010; Girard, 2022), the fact that participants were already engaged in positive environmental attitudes or behaviors introduced a bias that can play both ways, either increasing associations between exposure to art and influence on PEB variables, if participants were very positive and stimulated, or decreasing them, if participants considered that they learned or felt nothing new as a result of exposition to artworks, or that they were already engaged in PEB to the best of their capacity. In the reviewed studies, the second case was notably observed by Girard (2022) and Klöckner and Sommer (2021), where participants with already strong environmental attitudes showed less inclination to change. The authors were able to make this observation thanks to the establishment of baseline environmental profiles, thus reducing the uncontrolled bias. No author discussed the first occurrence although such a bias might have been present. In all cases, the establishment of baseline environmental profiles, as suggested above, can help future studies better prevent this bias.

Finally, all the reviewed studies showcased limitations in their data that prevented us from deepening our analysis of both emotions and PEB. While emotions were categorized as either positive or negative, there was no further exploration of the different types of emotions, which could have helped further understand what sort of emotions are activated by art. As an illustration of possible categorization, Martini et al. (2023) sorted out emotions that could affect intentions to adopt PEB in three categories: self-related emotions, social (pro-social) emotions, and pro-active, which could be more accurately renamed basic, self-conscious and cognitively complex emotions based on Reeve’s (2018) terminology. Martini et al. (2023) found cognitively complex emotions to exert the highest influence on the intention to behave in an environmentally responsible manner, as did Ma and Li (2024) when studying compassion. But other authors also found significant influence from basic emotions, most notably anger (e.g., Cho and Walton, 2009; Aguilar-Luzón et al., 2023), and self-conscious emotions, such as guilt (e.g., Nawijn and Biran, 2019) or pride (e.g., Russell et al., 2017).

Similarly, there was no differentiation between the different behaviors adopted or intended to be adopted by participants of the reviewed studies. Yet, different behaviors can be grounded in different motivations, and understanding these different motivations in future studies might help better unravel the processes behind the influence that art can exert on PEB. For example, López-García et al. (2025) differentiate between sustainable consumption and active participation, while Stern (2000) propose four categories of PEB: private-sphere PEB, environmental activism, non-activist public sphere PEB, and behaviors in organizations. The author further argues that different behaviors stem from different causes, with some that rely on context and individual capacities, or in other words, perceived behavioral control, while others rely more on personal attitudes. Differentiating types of emotions and PEB would certainly represent a step forward in understanding how art can support PEB adoption. The same idea could also be applied to art forms themselves. While only Klöckner and Sommer (2021) reported significant findings regarding the art forms, studying clearer links between participants profiles and the art forms that arouse the greatest emotional reaction could help further develop science-art initiatives.

## 6 Conclusion

This scoping review contributes to the literature by offering an exploration of PEB measurement tools in an artistic context. Based on this exploration, we propose recommendations for researchers interested in studying the influence of art on PEB, namely, to develop: a mixed-method approach, a literature-based conceptual basis, longitudinal studies, and baseline environmental profiles of participants. Further, we propose that providing Supplementary material and adopting a co-creation process might enhance the impact of science-art projects, although they might not be reflective of the day-to-day reality. We also offer a picture of how art influenced participants in different settings, as well as how these findings fit together in a greater picture. Notably, we show that art can play several roles that may ultimately influence PEB by acting primarily on intentions, attitudes and perceived behavioral control through emotional activation and by communicating environmental knowledge and awareness. This role, however, remains minimal without the establishment of greater societal and cultural norms in which diverse art forms can play their part to influence PEB. In doing so, we established that future research would need to develop a better understanding of the processes that determine how emotions influence the different variables of PEB, which in turn influence behavior. Art is certainly a promising avenue to help trigger emotions and unravel how the triggered emotions fit into the greater picture of civic engagement in PEB.

## Data Availability

The original contributions presented in the study are included in the article/Supplementary material, further inquiries can be directed to the corresponding author.
