# Supplementary material for: Influencing pro-environmental behaviors through visual arts: a scoping review of research designs and state of knowledge
Source: Front Psychol. 2025 Dec 4;16:1712588. doi: 10.3389/fpsyg.2025.1712588 (PMC12711806; doi:10.3389/fpsyg.2025.1712588)
Supplement: Supplementary file 1 [file Table_1.pdf]

SUPLEMENTARY TABLE A

Main characteristics of the studies included in this review

Study Reference	Art form and artwork	Study objectives	Sample					Survey Delivery Method(s)	Study Design
			Population	Age	Sex	Education	N		
Baldwin & Chandler (2010)	Festival, *Photovoice*	To understand the impact of photovoice on participants' awareness, understanding of others and inclination to take action in relation to climate change	Australia<br><br>Artists: 4<br>Residents: 8<br>Visitors: 4	Not specified			16	Post-workshop survey (onsite)	Mixed method (quantitative and qualitative)
Girard (2022)	Documentary film, *Cowspiracy, Demain, Découverte*	To understand the impact that climate change related documentaries can have on the adoption of pro-environmental behavior and verify the impact of different persuasion techniques used in those documentaries	Quebec, Canada<br><br>Adults	Average age was in the forties, all age groups were represented	Mostly women	Diverse, most participants were educated to university level	22	Pre- and post-surveys (onsite)<br><br>Follow-up 1 month after (online)	Mixed method (quantitative and qualitative)

Study Reference	Art form and artwork	Study objectives	Sample					Survey Delivery Method(s)	Study Design
			Population	Age	Sex	Education	N		
Hofman & Hughes (2018)	Documentary film, *The Sea & Me*	To explore whether the documentary, along with support materials provided post viewing, resulted in the adoption of conservation behaviors	Australia<br><br>Students in tourism degrees at the University of Queensland	Under 20: 16%<br>20-29: 81%<br>30+: 3%	75% female	Undergraduate: 64<br>Postgraduate: 118	182	Pre- and post-surveys (onsite)<br><br>Follow-up survey 70 days after viewing (online)	Quantitative
Howell (2011)	Movie, *The Age of Stupid*	To investigate the impact of the film on UK viewers' attitudes and behavior	United Kingdom<br><br>Moviegoers in the UK	16-24: 13%<br>25-34: 24.7%<br>35-44: 19.1%<br>45-54: 13%<br>55-64: 16%<br>65+: 13%<br>Not given: 1.2%	F: 56.2%<br>M: 40.1%<br>Not disclosed: 3.7%	First degree/masters: 68.5%<br>PhD: 9.9%<br>Lower qualifications: 14.8%<br>Not given: 6.8%	162	Pre- and post-viewing survey (onsite)<br><br>Follow up via online survey 10-14 weeks after viewing	Mixed method (quantitative and qualitative)
Klockner & Sommer (2021)	Exhibition, *ArtCOP21*	To research relations between psychological responses, artworks and the potential impact on	Paris, France<br><br>Visitors of the ArtCOP21	Mean: 36.6 years (SD = 16.4)	F: 56.2%<br>M: 43.6%	University: 71%<br>College or technical: 9.2%<br>High school: 15.9%<br>Primary school: 3.9%	883	Post-event survey	Quantitative

Study Reference	Art form and artwork	Study objectives	Sample					Survey Delivery Method(s)	Study Design
			Population	Age	Sex	Education	N		
		visitors in the form of support for pro-environmental policies							
Marks et al. (2014)	Festival, *Floating Land (FL)*	To evaluate the impact of the festival on the audience's intention to adopt or reinforce pro-environmental behaviors and explore whether the event contributed to developing their sense of place	Australia<br><br>(1) FL audience	16-25: 3<br>26-35: 9<br>36-45: 20<br>46-55: 28<br>56-65: 32<br>65+: 8	F: 60<br>M: 30	University: 58<br>Not university: 34<br>Other: 6	120	Post-event survey (onsite)	Mixed method (quantitative and qualitative)
			Australia<br><br>(2) FL workshops	Not specified			30	Post-workshop survey (onsite)	
			Australia<br><br>(3) Local residents	Not specified			36	Pre- and post-festival survey delivered in household letterboxes	
Schneller & Irizarry (2014)	Mural, *sea turtle murals*	To understand the impact of public art (sea turtle murals) on pro-environment behaviors related to protection of	Baja California Sur (B.C.S.), Mexico<br><br>Students and adults from	Students:<br>- min: 12<br>- max: 18<br>- mean: 14<br>Adults:<br>- min: 18<br>- max: 72<br>- mean: 39	F: 147<br>M: 175<br>Not disclosed: 11	NS	Total 333<br><br>Students 172<br>Adults 161	Semi-structured interviews	Qualitative

Study Reference	Art form and artwork	Study objectives	Sample					Survey Delivery Method(s)	Study Design
			Population	Age	Sex	Education	N		
		the marine environment	nine B.C.S. communities						
Sommer et al. (2019)	Immersive installation, *Pollution Pods (PP)*	To evaluate the impact of the exhibition on participants' intention to adopt and adoption of PEB	Trondheim, Norway<br><br>Visitors of Starmus Festival	Mean = 32.66	Average of 56% female with no significant difference among the 3 groups	University: 48%<br>College: 18%<br>High school: 25%<br>Primary school: 11%	1,016	Pre- and post-exhibition surveys (onsite)<br><br>Follow-up via a web platform to track carbon footprint (online)	Quantitative
			London, United Kingdom	Mean = 35.57		University: 79%<br>College: 15%<br>High school: 3%<br>Primary school: 3%	851		
			Comparison Groups	Mean = 38.33		Not specified	795		
