# Supplementary material for: Influencing pro-environmental behaviors through visual arts: a scoping review of research designs and state of knowledge
Source: Front Psychol. 2025 Dec 4;16:1712588. doi: 10.3389/fpsyg.2025.1712588 (PMC12711806; doi:10.3389/fpsyg.2025.1712588)
Supplement: Supplementary file 2 [file Table_2.pdf]

SUPPLEMENTARY TABLE B[1]

Categorization of Survey Measurement Items[2]

1. "At the water's edge": community voices on climate change (Baldwin & Chandler, 2010) (post-event survey)

Item	Emotional activation	Attitude	Intention	Perceived behavioral control	Behavior	Environmental awareness and knowledge	Other
Stimulated your curiosity or interest to find out more about climate change						x	
Raised your awareness about climate change issues						x	
Improved your understanding of others' perspectives about climate change						x	
Reinforced your existing views about climate change						x	
Shifted your views about climate change						x	
Encouraged you to take action on climate change		x					
Did you notice any differences between the three groups' perspectives?							x
Identify up to 3 photos that affected you or resonated most with you.	x						
To what extent has the Floating Land Festival as a whole affected your interest in and awareness of climate change issues?						x	
**Total: 9 items**	**11.1%**	**22.2%**	**0.0%**	**0.0%**	**0.0%**	**66.7%**	**11.1%**

[1] Only 7 of the 8 studies are included since Schneller & Irizarry (2014) used open-ended questions, which are not provided by the authors.
[2] Some items can be associated with two categories, hence the total percentage below the table, if calculated, can be above 100%.

2. Re-imagining the environment: using an environmental art festival to encourage pro-environmental behavior and a sense of place (Marks et al., 2014) (post-event survey)

**Item**	**Emotional activation**	**Attitude**	**Intention**	**Perceived behavioral control**	**Behavior**	**Environmental awareness and knowledge**	**Other**
Intended to change their environmental behavior as a result of attending the festival			x				
I am optimistic about environmental quality in the future		x					
A better environment starts with the individual		x					
It is normal to want to help the environment		x					
I feel guilty about the state of the environment	x						
Wished for *Floating Land* to continue							x
More proud of Boreen Point since *Floating Land*	x						
Felt influenced by *Floating Land*		x					
Felt more a part of the community since *Floating Land*	x						
Wanted to do more for the community since *Floating Land*			x				
Wanted to do more for the environment since *Floating Land*			x				
Felt influenced about the environment by *Floating Land*		x					
Total: 12 items	25.0%	41.7%	25.0%	0.0%	0.0%	0.0%	0.8%

3. Influence of persuasion techniques used in climate change documentaries on environmental behavior (Girard, 2022) (post-event and follow-up survey)

**Item**	**Emotional activation**	**Attitude**	**Intention**	**Perceived behavioral control**	**Behavior**	**Environmental awareness and knowledge**	**Other**
I know what the consequences of climate change are.						x	
Climate change is a topic that interests me.						x	
I seek out information on climate change phenomena regularly.					x		
I think changes in the climate are as serious as people say they are.						x	
I am worried about the consequences that climate change is having or will have in the future.	x						
Changes in the climate affect everyone.						x	
I don’t eat meat every week.					x		
I produce less or slightly more waste than my neighbors.					x		
I discuss climate change regularly.					x		
I automatically purchase pro-environmental products.					x		
I trust I can adopt more pro-environmental behavior.				x			
There may be several obstacles to adopting more pro-environmental behavior. If I really want to, I think I can overcome these obstacles.				x			
Overall, there are few events outside of my control that prevent me from changing my behavior.				x			
I intend to adopt more pro-environmental behavior.			x				

Item	Emotional activation	Attitude	Intention	Perceived behavioral control	Behavior	Environmental awareness and knowledge	Other
I am considering changing some of my life habits because of the issues highlighted in the documentary.			x				
The documentary made me change some of my behaviors because of the issues highlighted.					x		
I feel the documentary has made me more aware.						x	x
The documentary will have/probably had an influence on my everyday life.			x		x		
My behavior was challenged by the documentary.		x					
I identify with the documentary.	x						
I spoke to my family and friends about the documentary I watched.					x		
I didn't like the documentary.							x
I thought it was a high-quality documentary.							x
The documentary makes me optimistic about climate change.	x						
The documentary motivates or inspires me.	x						
The documentary makes me feel guilty.	x						
The documentary makes me sad.	x						
The documentary did not make me think about climate issues.						x	x
The documentary didn't make me reflect on my role in the climate situation.						x	x
The documentary challenges the rules of society and social norms.							x
I found the documentary impactful.							x
The documentary didn't make me want to see other documentaries.							x

Item	Emotional activation	Attitude	Intention	Perceived behavioral control	Behavior	Environmental awareness and knowledge	Other
Was there a moment in the documentary that you found more impactful? In a few words, which moment or moments were they?*							x
Did one or several moments in the documentary have an impact on you? Do you remember which moment or moments? Describe them in a few words.*		x					
Was there a moment in the documentary that you found unpleasant? In a few words, which moment or moments did you find unpleasant?	x						
**Total: 35 items**	**20.0%**	**5.7%**	**8.6%**	**8.6%**	**22.9%**	**20.0%**	**25.7%**

4. "Pollution Pods": The merging of art and psychology to engage the public in climate change (Sommer et al., 2019) (post-event survey)

Item	Emotional activation	Attitude	Intention	Perceived behavioral control	Behavior	Environmental awareness and knowledge	Other
To what extent does the artwork bring up each of these feelings within you? Happiness	x						
Guilt	x						
Hope	x						
Sadness	x						
A sense of awe	x						
Helplessness	x						
Inspiration	x						
Anger	x						
Surprise	x						
Anxiety	x						
Shame	x						
Pride	x						
Disgust	x						
Confusion	x						
Unaffected	x						
The artwork made me think about the problem of air quality in cities						x	
The artwork helped me understand the importance of people working together to make the world a more liveable place		x				x	
The artwork made me think about the living conditions of animals						x	
The artwork helped me see how actions we take today can have profound consequences for future generations		x				x	
The artwork made me think about environmental problems						x	
I gained a stronger sense of the connection between my actions and the well-being of people in other places						x	

Item	Emotional activation	Attitude	Intention	Perceived behavioral control	Behavior	Environmental awareness and knowledge	Other
The artwork made me think about the importance of long-term planning for the health of people		x				x	
The artwork is confrontational, i.e., has a shocking or aggressive undertone	x						
I gained a stronger sense of the connection between my actions and the health of the environment						x	
The artwork made me think and reflect on its meaning							x
The artwork made me think about the problem of climate change						x	
The artwork helped me see how actions can have profound unintended consequences for future generations		x				x	
The artwork made me think about life in developing countries						x	
The artwork made me more aware of the impact of my behavior on the environment						x	
The artwork made me think about the importance of long-term planning for the health of the planet		x				x	
I gained a strong sense of the consequences of the health of the environment for my health and well-being						x	
Every citizen must take responsibility for the environment		x					
Environmental problems generated in one country harm people all over the world		x					
The artwork seems relevant to my daily life							x
Ease of breathing is an important topic for me or my family							x
I feel partly responsible for the environmental problems on our planet		x					
The effects of environmental problems on public health are worse than we realize						x	

Item	Emotional activation	Attitude	Intention	Perceived behavioral control	Behavior	Environmental awareness and knowledge	Other
Over the next several decades, thousands of species will become extinct						x	
I feel that the main responsibility to take care of environmental problems lies with those that are affected by them		x					
I feel that it is my responsibility to do something to prevent climate change and other environmental problems		x					
The artwork highlights environmental problems that would affect me personally						x	
The topic of the artwork seems relevant to my daily life						x	
The balance in nature is delicate and easily upset						x	
We are approaching the limit of the number of people the earth can support						x	
Humans have the right to modify the natural environment to suit their needs		x					
If things continue on their present course, we will soon experience a major ecological catastrophe		x					
Humans were meant to rule over the rest of nature		x					
When humans interfere with nature, it often produces disastrous consequences		x					
Plants and animals have as much right as humans to exist		x					
The earth has plenty of natural resources if we just learn how to develop them		x					
Humans are severely abusing the environment		x					
The earth is like a spaceship with very limited room and resources		x					
The so–called “ecological crisis” facing humankind has been greatly exaggerated		x					

Item	Emotional activation	Attitude	Intention	Perceived behavioral control	Behavior	Environmental awareness and knowledge	Other
The balance of nature is strong enough to cope with the impacts of modern industrial nations		x					
Why is the climate system changing?						x	
Which of the following would you say is the main problem with rising levels of CO_2 in the atmosphere?						x	
What affects the climate system?						x	
How are air pollution and climate change linked?						x	
I intend to do something actively to help prevent environmental problems (continuously) in the future			x				
I intend to do something actively to help prevent climate change (continuously) in the future			x				
**Total items: 60**	**26.7%**	**33.3%**	**3.3%**	**0.0%**	**0.0%**	**40.0%**	**5.0%**

5. Protecting the Great Barrier Reef: analysing the impact of a conservation documentary and postviewing strategies on long-term conservation behaviour (Hofman & Hughes, 2018) (post-survey and follow-up)

Item	Emotional activation	Attitude	Intention	Perceived behavioral control	Behavior	Environmental awareness and knowledge	Other
We are part of the threat to marine environments						x	
There is a lot I can do to help protect marine environments				x			
We have a responsibility to leave healthy ecosystems for our families and future generations		x					
I want to do everything I can to protect and conserve marine environments		x					
I feel I need to help protect marine environments		x					
I feel I am a part of the solution to marine conservation problems		x					
I intend to reuse containers more often			x				
I am more likely to recycle			x				
I am more likely to conserve energy at home			x				
I am more likely to choose sustainable seafood			x				
I am more likely to increase my use of 'green' (non-plastic) shopping bags			x				
I intend to think about what I put down the drain (e.g., oil)			x				
I will probably talk to others about environmental issues			x				
I am more likely to purchase products that have minimal packaging			x				
I intend to make more effort to pick up other people's litter			x				
I am more likely to do volunteer work for a group that helps the environment			x				
I am more likely to participate in public land/water clean up activities			x				

Item	Emotional activation	Attitude	Intention	Perceived behavioral control	Behavior	Environmental awareness and knowledge	Other
I am more likely to look for information about the environment on social media (e.g., Facebook), on TV, in print or on the internet			x				
I am more likely to donate money to a nature or conservation organisation			x				
Being careful disposing down drains					x		
Recycling					x		
Picking up other people's litter					x		
Using 'green' (non-plastic) shopping bags					x		
Talking to others about environmental issues					x		
Actively purchasing products that have minimal packaging					x		
Looking for information about the environment on TV, in print or on the internet					x		
Conserving energy in the home					x		
Choosing sustainable seafood					x		
Participating in public land/water clean-up activities					x		
Donating money to a nature or conservation organisation					x		
Doing volunteer work for a group that helps the environment					x		
Reusing containers					x		
**Total items: 32**	**0%**	**12.5%**	**40.6%**	**3.1%**	**40.6%**	**3.1%**	**0.0%**

6. Lights, camera... action? Altered attitudes and behavior in response to the climate change film *The Age of Stupid* (Howell, 2011)

Item	Emotional activation	Attitude	Intention	Perceived behavioral control	Behavior	Environmental awareness and knowledge	Other
Concern about climate change						x	
I feel motivated to try to do something about climate change/global warming		x					
I can do something to prevent climate change/global warming getting worse				x			
I know what I can do to reduce my carbon emissions				x			x
Cutting my carbon emissions won't make a difference to the problem of climate change/global warming		x					
It's worth lobbying politicians about climate change/global warming		x					
I do as much as I can about climate change/global warming					x		
I fear humanity will not do enough to prevent catastrophic climate change/global warming		x					
How likely do you think it is that the world could be devastated by climate change/global warming and related problems, in the way it is in the film, by 2055?						x	
What message are you taking away from the film?							x
Questions about specific beahaviors. Trying to raise awareness among people I know					x		
Sent message to politician(s) - last 12 months					x		
Actively involved in campaigning group					x		
Attended rally outside Scottish parliament					x		
Calculated 'carbon footprint' - last 12 months					x		
Installed low energy light bulbs – most/all lights					x		
Turned down heating/cut time heating is on					x		

Item	Emotional activation	Attitude	Intention	Perceived behavioral control	Behavior	Environmental awareness and knowledge	Other
Washing clothes at 30°C (usually/always)					x		
Drying clothes on rack (usually/always)					x		
Installed more insulation/draught-proofing					x		
Changed to ‘green electricity’ supplier/tariff					x		
Generating energy through home renewables					x		
Cut down/avoid driving					x		
Car sharing/car club (leave blank if never drive)					x		
Planning/taking holidays without flying this year					x		
Decided to reduce/stop holiday flying long-term					x		
Avoiding buying bottled water					x		
Buying more local produce					x		
Reduced meat consumption/eat vegetarian/vegan					x		
Composting food waste					x		
Barriers to action. Cost				x			
Lack of options				x			
Lack of information				x			
Inconvenience/discomfort				x			
Lack of time				x			
No point at the moment				x			
Other				x			
**Total items: 37**	**0.0%**	**10.8%**	**0.0%**	**24.3%**	**56.8%**	**5.4%**	**5.4%**

7. Visual art inspired by climate change – An analysis of audience reactions to 37 artworks presented during the 21st UN Climate Summit in Paris (Klöckner & Sommer, 2021) L. K.

Item	Emotional activation	Attitude	Intention	Perceived behavioral control	Behavior	Environmental awareness and knowledge	Other
The artwork appears to be of considerable artistic quality							x
happiness	x						
hope	x						
inspiration/enthusiasm	x						
anger	x						
anxiety	x						
sadness/disappointment that nothing is happening to prevent climate change	x						
if they imagined the artist to be someone like themselves							x
if they imagined the artist to be someone with values similar to themselves							x
if they imagined the artist to be someone expressing the values of the public							x
The artwork makes me think and reflect on its meaning							x
the artwork seems relevant to my daily life							x
the artwork highlights the consequences of climate change that would affect me personally						x	
the artwork makes me think about the problem of climate change						x	
the artwork makes me think about my own role within the current climate situation		x					
the artwork makes me more aware of my behavior's impact on the environment						x	
How important is it to you that climate change and the environment are given a high priority in policymaking?		x					
**Total items: 17**	**35.3%**	**11.8%**	**0.0%**	**0.0%**	**0.0%**	**17.6%**	**35.3%**
