# Supplementary material for: Influencing pro-environmental behaviors through visual arts: a scoping review of research designs and state of knowledge
Source: Front Psychol. 2025 Dec 4;16:1712588. doi: 10.3389/fpsyg.2025.1712588 (PMC12711806; doi:10.3389/fpsyg.2025.1712588)
Supplement: Supplementary file 3 [file Data_Sheet_1.DOCX]

Appendix A

**Search chains**

1. Web of Science

(((((TS=(artist* OR arts OR documentar* OR theat* OR museum OR museal OR eco-visuali* OR exhibition* OR cinema OR movie* OR film OR festival* OR photo*)) AND TS=(pro-environment* OR environmentally OR sustainable OR "climate change")) AND TS=(behavio* OR attitude* OR action*))) AND AB=(survey* OR questionnaire* ))

1. Érudit Database

(Titre, résumé, mots-clés : artiste* OU arts OU documentaire* OU théâtre OU muséal OU musée OU éco-visualisation* OU cinéma OU film* OU festival* OU photo* OU "art environnemental") ET (Titre, résumé, mots-clés : écoresponsable* OU pro-environnement* OU écologique* OU environnementa* OU "changements climatiques") ET (Titre, résumé, mots-clés : comportement* OU attitude* OU action*) ET (Tous les champs : questionnaire* OU entrevue*) ET (Publié entre 2001 et 2024) ET (Fonds : ['Érudit', 'UNB', 'Persée', 'FRQ'])
